# Reduction in Preterm Preeclampsia after Contingent First-Trimester Screening and Aspirin Prophylaxis in a Routine Care Setting

**DOI:** 10.3390/diagnostics12081814

**Published:** 2022-07-28

**Authors:** Cristina Trilla, Josefina Mora, Nuria Ginjaume, Madalina Nicoleta Nan, Obdulia Alejos, Carla Domínguez, Carmen Vega, Yessenia Godínez, Monica Cruz-Lemini, Juan Parra, Elisa Llurba

**Affiliations:** 1 Department of Obstetrics and Gynaecology, Institut d’Investigació Biomèdica Sant Pau-IIB Sant Pau, Hospital de la Santa Creu i Sant Pau, 08025 Barcelona, Spain; ctrilla@santpau.cat (C.T.); nuriag544@gmail.com (N.G.); oalejos@santpau.cat (O.A.); cdominguezg@santpau.cat (C.D.); mvega@santpau.cat (C.V.); yessp77@gmail.com (Y.G.); mcruzl@santpau.cat (M.C.-L.); jparra@santpau.cat (J.P.); 2 Department of Medicine, Universitat Autònoma de Barcelona, 08193 Barcelona, Spain; jmora@santpau.cat; 3 Primary Care Interventions to Prevent Maternal and Child Chronic Diseases of Perinatal and Developmental Origin Network (RICORS, RD21/0012/0001), Instituto de Salud Carlos III, 28029 Madrid, Spain; 4 Biochemistry Department, Hospital de la Santa Creu i Sant Pau, 08025 Barcelona, Spain; mnan@santpau.cat

**Keywords:** preeclampsia screening, first trimester, placental growth factor, low-dose aspirin, obstetric outcome, clinical practice

## Abstract

**Objectives:** Several multivariate algorithms for preeclampsia (PE) screening in the first trimester have been developed over the past few years. These models include maternal factors, mean arterial pressure (MAP), uterine artery Doppler (UtA-PI), and biochemical markers (pregnancy-associated plasma protein-A (PAPP-A) or placental growth factor (PlGF)). Treatment with low-dose aspirin (LDA) has shown a reduction in the incidence of preterm PE in women with a high-risk assessment in the first trimester. An important barrier to the implementation of first-trimester screening is the cost of performing tests for biochemical markers in the whole population. Theoretical contingent strategies suggest that two-stage screening models could also achieve high detection rates for preterm PE with lower costs. However, no data derived from routine care settings are currently available. This study was conducted to validate and assess the performance of a first-trimester contingent screening process using PlGF for PE, with prophylactic LDA, for decreasing the incidence of preterm PE. **Methods:** This was a two-phase study. In phase one, a contingent screening model for PE was developed using a multivariate validated model and a historical cohort participating in a non-interventional PE screening study (n = 525). First-stage risk assessment included maternal factors, MAP, UtA-PI, and PAPP-A. Several cut-off levels were tested to determine the best screening performance, and three groups were then defined (high-, medium-, and low-risk groups). PlGF was determined in the medium-risk group to calculate the final risk. Phase two included a validation cohort of 847 singleton pregnancies prospectively undergoing first-trimester PE screening using this approach. Women at high risk of PE received prophylactic treatment with 150 mg of LDA. The clinical impact of the model was evaluated by comparing the incidence of early-onset (<34 weeks) and preterm (<37 weeks) PE between groups. **Results:** Cut-off levels for the contingent screening model were chosen in the first and second stages of screening to achieve a performance with sensitivities of 100% and 80% for early-onset and preterm PE detection, respectively, with a 15% false positive rate. In the development phase, 21.5% (n = 113) of the women had a medium risk of PE and required second-stage screening. In the prospective validation phase, 15.3% (n = 130) of the women required second-stage screening for PlGF, yielding an overall screen-positive rate of 14.9% (n = 126). The incidence of preterm PE was reduced by 68.4% (1.9% vs. 0.6%, *p* = 0.031) after one year of screening implementation. **Conclusions:** Implementation of contingent screening for PE using PlGF in a routine care setting led to a significant reduction (68.4%) in preterm PE, suggesting that contingent screening can achieve similar results to protocols using PlGF in the whole population. This could have financial benefits, with a similar reduction in the rate of preterm PE.

## 1. Introduction

First-trimester screening for early-onset preeclampsia (PE) was included in the Guideline for Pregnancy Follow-up in Catalonia in 2018 [1]. According to this guideline, universal screening for PE should be performed for all pregnant women, using a combination of maternal and obstetric factors, mean arterial pressure (MAP), uterine artery Doppler (UtA-PI), and pregnancy-associated plasma protein-A (PAPP-A). Low-dose aspirin (LDA) should then be offered to high-risk women until 36 weeks of pregnancy, based on the results of the Aspirin versus Placebo in Pregnancies at High Risk for Preterm Preeclampsia (ASPRE) trial [2]. However, implementation of a universal screening protocol involving multivariate models is challenging in clinical practice, and specific training and quality programs are required.

Various multivariate screening models for PE have been developed in the past few years [3,4,5], yielding similar detection rates for early-onset and preterm PE. These programs consist of different combinations of maternal, biophysical, and biochemical factors and are based on different statistical methodologies. Regarding biochemical markers, models incorporating PAPP-A are potentially easier to implement without a substantial increase in costs, since PAPP-A is already available from aneuploidy screening. In contrast, models including placental growth factor (PlGF), a pro-angiogenic involved in placental development [6], have demonstrated superior detection rates for PE compared to algorithms including PAPP-A alone [7], although these models entail additional costs. Thus, the additional cost of determining PlGF can be a significant barrier to universal screening implementation.

As an alternative to models including all the above mentioned variables, contingent screening strategies have been suggested, with two studies evaluating the theoretical performance of two-stage protocols [8,9]. Both studies concluded that such strategies are feasible and can achieve similar detection rates to those of models including all variables, representing potential financial savings. Despite these promising results, no data derived from prospective studies are available regarding implementation and results in clinical practice. The aim of our study was to evaluate the clinical impact of a contingent screening program to prevent preterm PE after one year of its implementation. We also sought to describe the development process of this model and to identify the challenges for its clinical implementation.

## 2. Materials and Methods

This study was a population-based study conducted at the Hospital de la Santa Creu i Sant Pau University Hospital, Barcelona, Spain. First- and second-trimester ultrasounds of pregnant women within the hospital’s area of influence were performed at the hospital. This study was approved by the Ethics Committee of the Institutional Review Board, and all the women gave their informed consent. This is a pilot study of a larger trial on contingent screening for PE, registered with ClinicalTrials.gov (NCT04767438).

### 2.1. Study Design: Model Development

This study consisted of two phases. In both phases, women were included at the time of the first-trimester ultrasound. The first phase involved a historical cohort of women with singleton pregnancies participating in a prospective PE screening protocol, recruited between July 2016 and November 2018, with serum samples stored for the study. Pregnant women living in our hospital referral region had the first- and second-trimester scans at our institution. All women with a singleton pregnancy attending for their first-trimester ultrasound were invited to participate. Thus, the study group included both low- and high-risk women. Data on pregnancy outcomes were analyzed retrospectively, and variables from the first-trimester ultrasound were used for the model development. The first-trimester risk of PE was thus retrospectively calculated using a multivariate Gaussian distribution model validated in our population [4]. In a first stage, maternal factors (ethnicity, weight, height, smoking status, parity, history of PE, pre-existing diabetes, hypertension, thrombophilia, renal or autoimmune conditions), biophysical markers (MAP and UtA-PI), and PAPP-A were included, giving a preliminary risk of PE. In the second stage, PlGF was determined and added to the predictive model. Screening performance for early and preterm PE was analyzed at a fixed false positive rate (FPR) of 15%, following the methodology described elsewhere [4]. The final model was defined including three risk groups for PE in the first stage of screening: (1) risk ≥ 1/50, high-risk group with no further testing, (2) risk between 1/51 and 1/500, medium-risk group requiring further testing, and (3) risk ≤ 1/501, low-risk group. For the second stage, the serum concentrations of PlGF of the medium-risk group were considered, giving the final risk of PE at a cut-off value of 1/160. Given the historical nature of the cohort and the fact that sample size was limited to patient and serum sample availability, the final model and cut-off levels of both stages were chosen to obtain a final screen-positive rate between 15 and 20% for this cohort, with a fixed false positive rate (FPR) of 15%.

In this population, prophylactic treatment with LDA (100 mg) was given based on maternal factors alone, according to our institutional protocol for PE, updated in 2016. Therefore, patients were offered treatment in the following situations: (1) the presence of one major risk factor, i.e., previous PE, renal disease, antiphospholipid syndrome, systemic lupus erythematosus, diabetes, or hypertension or (2) two or more moderate risk factors such as pregnancy from egg-donation treatment, maternal age over 40, nulliparity or an intergenesic period of >10 years, family history of PE, or multiple gestation.

### 2.2. Study Design: Validation Cohort

**Figure 1:**
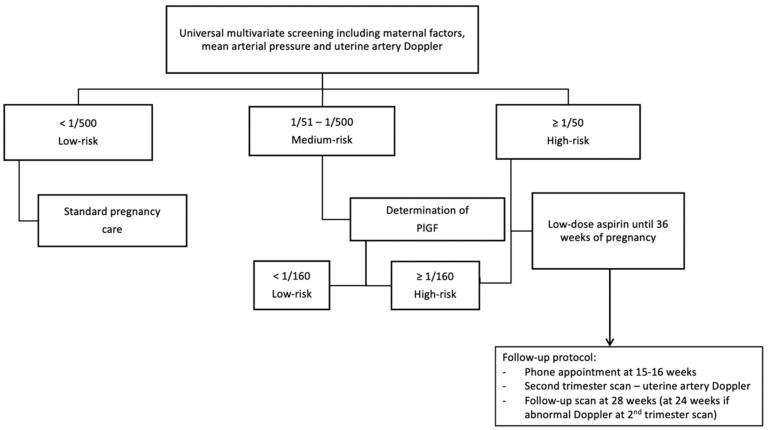
Screening protocol and follow-up strategy for women at high risk of developing preeclampsia.

The second phase was a prospective population-based study conducted between 1 February 2019 and 28 February 2020. All sonographers performing first-trimester measurements underwent specific training and accreditation by the Fetal Medicine Foundation for PE screening for this phase. Women with a singleton pregnancy attending for a first-trimester ultrasound were invited to participate. PE risk assessment was performed according to the previously described contingent model. The list of women requiring PlGF determination was provided to the Biochemistry Department on a weekly basis, on Fridays. PlGF determinations were performed the following Monday, with results available on Tuesday. A phone call appointment was scheduled one week after first-stage screening, to provide final information on results and counseling. Patients with a high risk of PE were offered prophylactic treatment with 150 mg LDA at bedtime from the moment of screening until 36 weeks. If the women refused the second stage of screening, a cut-off value of 1/150 was used for defining high-risk women and offering treatment. This cut-off was chosen to preserve the performance of the screening in the event of women refusing a two-stage approach. A specific follow-up protocol for high-risk pregnancies was established, which included telephone follow-up within three weeks of screening to assess tolerance and adherence to treatment. Adherence was verbally reassessed at every appointment with the midwife or physician. An additional ultrasound was scheduled at 28 weeks to assess fetal growth and UAt-PI. Figure 1 shows the screening and follow-up protocols. An email address was provided to all high-risk patients for online consultation, to enhance access to timely advice and service if needed.

### 2.3. Sample Collection and Laboratory Procedures

Serum samples remaining from first-trimester routine analysis were stored for the study. Serum was separated by centrifugation at 1500× *g* for 10 min at 4 °C, and samples were stored at −80 °C until they were analyzed. Serum concentrations of PlGF and PAPP-A were determined by fully automated electrochemiluminescence immunoassays, using a Cobas e 601 analyzer (Roche Diagnostics). Multiples of the median (MoM) values for PAPP-A and PlGF, calculated from locally derived normal medians using the above-mentioned multivariate Gaussian distribution model, were considered for analysis.

### 2.4. Data Collection

Maternal and pregnancy characteristics were prospectively recorded at the time of recruitment and confirmed after delivery by individual chart review. Gestational age was determined according to fetal crown–rump length (CRL) in all cases. Data from the first-trimester ultrasound were collected, i.e., gestational age, CRL, and UAt-PI, measured transabdominally. Blood pressure (BP) was measured once in one arm (right or left, without distinction), after a 5 min rest with women seated, at the time of the first-trimester ultrasound, according to our current clinical practice. A calibrated Tensoval Duo Control (Hartmann AG, 89,522 Heidenheim, Germany) was used. The MAP was calculated as diastolic BP + (systolic—diastolic BP)/3. BP was measured a single time in one arm. Exclusion criteria were major anatomic malformations, confirmed chromosomal or genetic abnormalities, and second-trimester pregnancy loss. Since recent evidence suggests that SARS-CoV-2 infection during pregnancy might increase the risk of PE, women with SARS-CoV-2 infection were also excluded, to avoid possible confounding effects [10].

Preeclampsia was defined according to the guidelines of the International Society for the Study of Hypertension in Pregnancy [11]. Thus, diagnosis was based on systolic BP ≥ 140 mmHg or diastolic BP ≥ 90 mmHg on repeated occasions after 20 weeks’ gestation and proteinuria (dipstick urinalysis ≥ 1+ or protein/creatinine ratio ≥ 30 mg/mmol (0.3 mg/mg)) or another maternal organ dysfunction. All cases of PE were classified according to the updated definition of PE in the 2018 Guideline [11], including those of the historical cohort. PE was classified according to gestational age at delivery as early-onset (<34 weeks), preterm (<37 weeks), or term (≥37 weeks). Birth-weight centiles were calculated according to local standards [12]. Small for gestational age (SGA) age was defined as a birth weight below the 10th centile. Abruptio placentae was defined as premature separation of the placenta after 20 weeks’ gestation.

### 2.5. Statistical Analysis

Statistical analyses for both model development and validation were performed using the IBM-SPSS software program, v.26.0 (IBM-SPSS Inc., Chicago, IL, USA). Upon development of the model, it was incorporated into the SsdwLab6 version 6.1 package (SBP Soft 2007 S.L.), which is the prenatal program used for aneuploidy screening at our hospital. The diagnostic performance of the contingent screening model in the historical cohort was characterized by determining sensitivity, specificity, screen-positive rate, positive predictive value (PPV), and negative predictive value (NPV) for early and preterm PE, with the chosen cut-off points, at a fixed FPR of 15%. MoM biomarker equations and the predictive algorithm were based on the previously published model [4], since this model has already been validated in the same population. However, MoM analysis was also performed in the control group (no PE group) for internal validation for model development.

Pregnancy outcomes of the two study groups were compared to assess the clinical impact of the screening program. Data are shown as the mean (standard deviation, SD) or percentages for continuous and categorical variables, respectively, and the median (interquartile range, IQR) for non-parametric variables. Associations between variables were evaluated using Student’s *t*-test, the χ^2^ test, or the Mann–Whitney *U* test, where appropriate. All reported *p*-values were 2-sided, and unequal variances were assumed. A *p*-value of < 0.05 was used to define statistical significance.

## 3. Results

**Figure 2:**
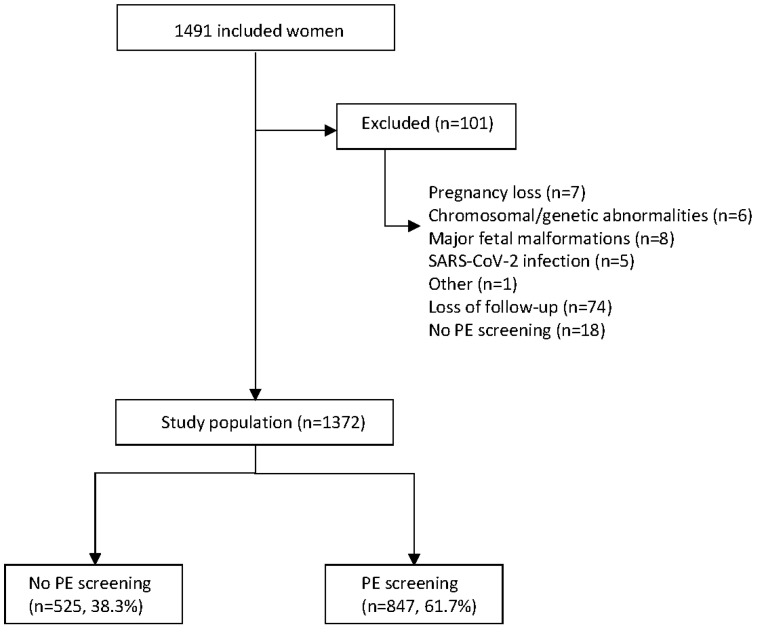
Flowchart for the study population.

A total of 1491 women were included in the study. Of these, 1390 met the inclusion criteria. The historical group included 525 women (38.3%) and the prospective validation group included 847 women (61.7%). Figure 2 depicts the flowchart for the study.

The maternal and pregnancy characteristics and the biochemical and biophysical variables of the study population are presented in Table 1. Women included in the prospective group had a higher BMI (23.5 vs. 24.2, *p* = 0.007) and higher rates of pregnancies achieved through assisted reproductive technologies (6.7% vs. 10.4%, *p* = 0.020). More-over, there were some differences in biochemical and biophysical markers between groups, with women of the prospective group having lower PAPP-A levels and a lower mean UAt-PI.

**Figure 3:**
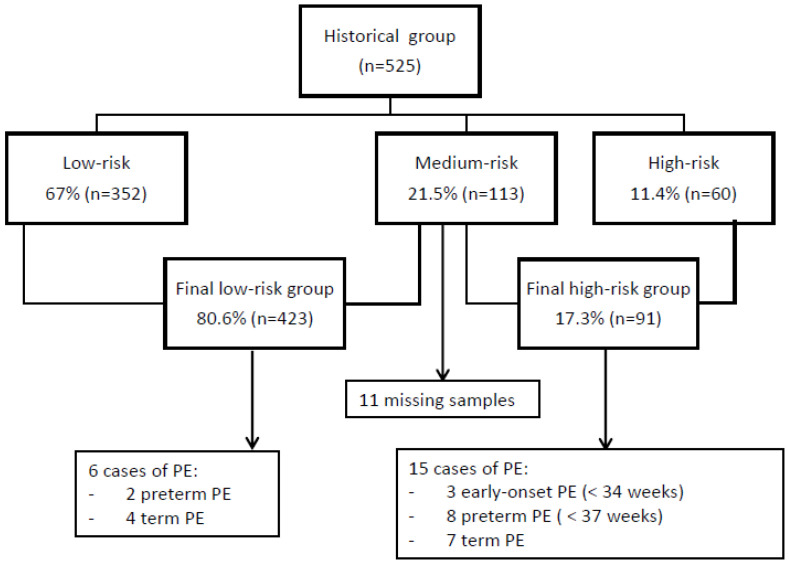
Screening results and obstetric outcomes of the historical group. PE, preeclampsia.

Figure 3 shows the screening results and the incidence of PE for the historical group. At the first stage of screening, 11.4% (n = 60) of the women had a high risk of PE. The medium-risk group included 21.5% (n = 113) of the women, and PlGF was determined in 102 women. The final high-risk group included 91 women (17.3%). The overall incidence of PE was 4% (n = 21). Of those, 47.6% (n = 10) were preterm cases and 52.4% (n = 11) occurred at term. The high-risk group according to contingent screening included 80% of the preterm PE cases and all cases of early-onset PE. Thus, for early-onset PE, sensitivity, specificity, PPV, and NPV were 100%, 85%, 4%, and 100%, respectively, for an FPR of 15%. For preterm PE, sensitivity, specificity, PPV, and NPV were 80%, 85%, 9%, and 99%, respectively, for an FPR of 15%. The positive-screen rate was 17.3%. In this group, 1.9% (n = 10) of the women were given LDA at 100 mg.

**Figure 4:**
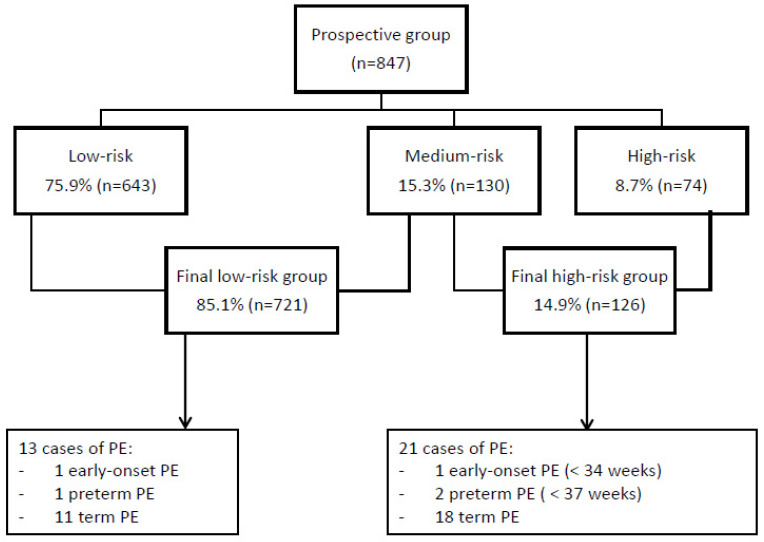
Screening results and obstetric outcomes of the prospective group. PE, preeclampsia.

Figure 4 shows the screening results and incidence of PE for the prospective group. At the first stage of screening, 8.7% (n = 74) of the women had a high-risk result and 15.3% (n = 130) required second-stage screening with PlGF. The final positive-screen rate was 14.9% (n = 126). Of those, 84.9% (n = 107) received treatment with LDA at 150 mg at bedtime, until 36 weeks. Assessment within 3 weeks of treatment initiation showed that LDA was not taken according to the protocol in 19 cases, due to adverse effects or intolerance (n = 4), prescription of a lower dose of aspirin (n = 5), allergy to aspirin or medical contraindication (n = 4), patient refusal of treatment (n = 5), or delay in final screening result (n = 1). Three of these women (15.7%) developed term PE. Women were contacted and informed of their final risk of PE according to the protocol in all cases except one where the telephone appointment was not scheduled in a timely way, and in this case, second-stage screening was performed after 16 weeks, yielding a high-risk result. This woman developed term PE. Six women (0.7%) refused second-stage screening, and here a cut-off value of 1/150 was used to determine high risk. Pregnancy follow-up of high-risk women was performed according to the protocol in all cases. Quality assessment for UAt-PI was performed for all sonographers. Internal validation of the model was performed with MoM analysis of biochemical markers of the control group (no PE group), showing suitable results as follows: PAPP-A MoM 1.04 (0.71–1.49) and PlGF MoM 0.99 (0.76–1.34). MoM analyses of the PE patients gave values of 0.87 (0.55–1.30) and 0.90 (0.59–1.23) for PAPP-A and PlGF, respectively.

The obstetric outcomes are summarized in Table 2. The incidence of preterm PE (below 37 weeks) was reduced significantly by 68.4% after implementation of the contingent screening model (1.9% before vs. 0.6% after, *p* = 0.031). In contrast, the rate of term PE increased by 61.9%, although these results were not statistically significant. There were two cases of early-onset PE in the prospective group. The first patient was classified as high risk, and LDA was prescribed according to the protocol, with optimal adherence to treatment. However, this patient developed severe early-onset PE with fetal growth restriction and required delivery at 31^+6^ weeks. The second patient was classified as low risk but developed severe early-onset PE with fetal growth restriction and required delivery at 33^+1^ weeks. The rate of abruptio placentae was also significantly decreased with prospective screening (0.8% before vs. 0% after, *p* = 0.021).

## 4. Discussion

Our study suggests that implementation of contingent screening for PE in clinical practice, using PlGF in 15% of the population, can achieve a significant decrease in preterm PE.

Since publication of the ASPRE trial [2], the need for first-trimester screening programs for PE has become urgent for obstetricians. However, although awareness of LDA treatment exists, and its effectiveness is unarguable [13], significant concerns persist regarding screening implementation in clinical practice. Firstly, the choice of the screening algorithm will depend on the characteristics of the population to be screened [14]. Secondly, the choice regarding biochemical variables is debatable [7,15], and the balance between screening performance and costs must be considered.

The performance of multivariate screening models is superior to methods based on maternal risk factors alone [16,17]. Recently, the FIGO Working Group on Good Clinical Practice in Maternal–Fetal Medicine endorsed first-trimester screening in singleton pregnancies, using a combination of maternal factors, UAt-PI, MAP, and PlGF at 11–13 weeks [18]. In our study, PE screening was implemented with the same software used for aneuploidy screening. This decision facilitated the integration of results and clinical implementation of the program. Moreover, this screening algorithm had been validated in our po-pulation. [4]

The model validated in our population was developed using both PAPP-A and PlGF. Higher performance of the model was obtained using PlGF than using PAPP-A, but both models are currently available in Catalonia, and screening can currently be performed using either PAPP-A or PlGF. Acknowledging that PlGF is the preferred biomarker for PE screening [7], we developed a contingent model using a historical population. Research regarding two-stage screening for PE has been conducted from a theoretical perspective. The results focused on screening performance rather than clinical aspects of implementation [8,9]. In our study, we found that a contingent model can be implemented with well-established protocols regarding methodology, quality assessment, patient counseling, and pregnancy follow-up. We consider it important that all these aspects are carefully consi-dered in advance when implementing any screening protocol. There was a high acceptance of the two-stage model, with only six women refusing the second stage of screening. For the medium-risk group, results were available within one week after the first assessment. Since the initiation of LDA prophylaxis is time-sensitive [19], it is crucial to establish an itinerary for the results, to avoid delays in obtaining the final risk assessment.

We found a significant reduction of preterm PE after one year of prospective screening, determining PlGF in 15% of our population. Previous contingent models tested se-veral algorithms to achieve a 70% detection rate for preterm PE. However, in these algorithms, PlGF had to be determined in 30–40% of the population [8,9], which entails higher costs. In our setting, UAt-PI and MAP were easily incorporated at the time of the first-trimester ultrasound, and PAPP-A was available from aneuploidy screening. Likewise, maternal factors were assessed at the first appointment. Thus, these variables were included in the first stage, and PlGF was reserved for the medium-risk group only. From a pragmatic viewpoint, this algorithm is easier to implement than determining UAt-PI along with PlGF up to the second stage. More importantly, despite the limited sample size included in our study, the reduction in preterm PE incidence was consistent with previous research [2]. Interestingly, our results are also consistent with previous data showing that LDA may delay the onset of the disease, since late-onset PE rates increased after the implementation of screening [20].

The aim of the study was not to assess the effectiveness of aspirin in decreasing the incidence of preeclampsia, as this had already been demonstrated in the ASPRE trial, but to evaluate the clinical impact of a universal contingent screening protocol. The reduction in preterm PE observed by comparing perinatal outcomes of the historical and prospective groups corroborate the fact that the screening protocol resulted in good selection of high-risk women who might benefit from the treatment under clinical practice conditions. Regarding perinatal outcomes, we also found a reduction in placental abruption in the treated group. These results are concordant with previous research conducted on that topic [21], although the numbers were too small to drive meaningful conclusions. No differences were found in the rate of SGA between groups. Overall, and despite baseline characteristics seeming to favor worse outcomes in the validation group, contingent screening was able to detect high-risk cases where LDA prophylaxis may have been of benefit.

Our contingent model was developed for an FPR of 15%, while previous studies adopted an FPR of 10%. Since the model was defined using a retrospective cohort, we decided to use a higher FPR to achieve high detection rates. The screen-positive rate was 17.3% in the historical group and 14.9% in the prospective group. The fact that the UAt-PI was significantly lower in the prospective group could explain this difference. However, although the choice of the FPR and cut-offs is always arbitrary, we acknowledge that some modifications in this model may be necessary to reduce the screen-positive rate without affecting the clinical results.

Regarding the variables included in the screening model, the selection of the biochemical markers represents an important decision. Models including PlGF have been shown to perform slightly better than models using PAPP-A [7]. Nevertheless, models using PAPP-A have demonstrated good detection rates without the added cost of determining PlGF in all women [15]. Moreover, although models using PlGF are cost-effective compared with screening using maternal factors alone [22], no studies have compared models using PAPP-A alone, PlGF alone, or PlGF in a subgroup of the population in a contingent protocol. Further research is needed to confirm which screening strategy has the best cost-effectiveness profile in different populations.

Our study has some limitations. This was a consecutive cases cohort study, based on the availability of patients, and lacked power calculation. The historical cohort used to develop the model included only three cases of early-onset PE, and this could impact the performance of the screening. In addition, quality assessment of the UAt-PI was not performed in the historical cohort. To address these limitations and avoid under-detection of high-risk women, the model was defined with a 15% FPR. Furthermore, contingent screenings have potential limitations in terms of clinical follow-up compared with one-step strategies. As demonstrated in our study, the development of specific itineraries for final risk assessment is crucial to avoid missing results. Finally, the prophylactic dose of aspirin used in the historical cohort was 100 mg, following the recommendations of our institutional protocol at that time. Only 1.9% of women were given 100 mg of aspirin in this group. The dose of aspirin was increased to 150 mg in the prospective phase, following the recommendations of the ASPRE trial and the Guideline for Pregnancy Follow-up implemented in Catalonia in 2018. We acknowledge that developing a screening model based on a historical group has significant limitations, but we estimate that the use of a lower dose of aspirin in only a small proportion of women from this group was a factor that, precisely, allowed a more accurate development of the model.

Despite these limitations, our study also had various strengths. Firstly, the original screening model for PE had already been validated in our population. This is important since performance may vary depending on the characteristics of the population to be screened [23,24,25]. Secondly, this study was conducted in the context of current clinical practice, which enhances the external validity of the results. Thirdly, describing the me-thodology used for clinical implementation of a multivariate contingent screening process for PE could be of value in clinical settings where universal PlGF is not feasible.

To conclude, the implementation of contingent screening for PE in clinical practice is feasible with well-defined protocols. Contingent screening determining PlGF in a medium-risk group can achieve similar results to protocols using PlGF in the whole population. Further studies involving larger populations are required to confirm our findings.

## Figures and Tables

**Table 1:** Baseline characteristics of study participants according to study groups.

Maternal and Pregnancy Characteristics	Retrospective Screening (n = 525)	Prospective Screening (n = 847)	*p*
General characteristics *			
Maternal age (years)	33.2 (4.7)	33.7 (5.2)	0.062
Body mass index (kg/m^2^)	23.5 (4.1)	24.2 (4.4)	0.007
Smoking during pregnancy	36 (6.9)	38 (4.5)	0.065
Ethnicity			0.012
Caucasian	398 (75.8)	563 (66.7)	
Latin-American	90 (17.1)	193 (22.9)	
Asian	13 (2.5)	20 (2.4)	
Afro-Caribbean	7 (1.3)	18 (2.1)	
North African	10 (1.8)	24 (2.8)	
Other	8 (1.5)	26 (3.1)	
Medical history *			
Chronic hypertension	3 (0.6)	13 (1.5)	0.078
Thyroid condition	39 (7.2)	78 (9.2)	0.231
Diabetes mellitus	5 (0.9)	4 (0.5)	0.316
Autoimmune condition	3 (0.6)	11 (1.3)	0.271
Neurologic condition	1 (0.2)	13 (1.5)	0.023
Thrombophilia	8 (1.5)	9 (1.1)	0.461
Renal disease	1 (0.2)	3 (0.4)	>0.999
Pregnancy history *			
Nulliparous	314 (59.8)	485 (57.3)	0.368
Previous PE	10 (1.9)	19 (2.2)	0.847
Previous SGA	28 (5.3)	38 (4.5)	0.517
Repeated miscarriage	29 (5.5)	32 (3.8)	0.139
ART conception *	35 (6.7)	88 (10.4)	0.020
*Biophysical markers*			
MAP (mmHg) ^†^	83.6 (8.5)	83.3 (7.7)	0.396
MAP (MoMs) ^†^	1.03 (0.95–1.11)	1.03 (0.96–1.11)	0.540
Mean UAt-PI ^†^	1.74 (1.43–2.06)	1.68 (1.38–1.99)	0.028
Mean UAt-PI (MoMs) ^†^	1.14 (0.94–1.37)	1.09 (0.91–1.30)	0.005
*Biochemical markers*			
GA at blood sampling (weeks) *	10.6 (1.1)	10.6 (1.1)	0.662
PAPP-A (MoMs) ^†^	1.08 (0.73–1.55)	1.01 (0.69–1.43)	0.029
PlGF (MoMs) ^†^	0.98 (0.70–1.39)	0.99 (0.73–1.26)	0.327
*GA at screening* (weeks)	12.9 (0.6)	12.8 (0.6)	0.715
CRL (mm)	65.6 (7.7)	65.5 (8.0)	0.773

* Data are given as mean (SD) or n (%). ^†^ Data are given as median (interquartile range, IQR). PE: preeclampsia; SGA: small for gestational age; ART: assisted reproductive technologies; MAP: mean arterial pressure; UAt-PI: uterine artery pulsatility indices; GA: gestational age; PAPP-A: associated plasma protein-A; PlGF: placental growth factor; MoMs: multiples of the median; CRL: crown–rump length.

**Table 2:** Obstetric outcomes according to study groups.

	Retrospective Group (n = 525)	Prospective Group (n = 847)	*p*
*Pregnancy treatments*			
Aspirin	10 (1.9)	117 (13.8)	<0.001
LMWH	12 (2.3)	19 (2.2)	>0.999
*Birth characteristics* *			
GA at birth (weeks) *	39.7 (2.3)	39.6 (1.9)	0.648
Female gender	268 (51.1)	410 (48.5)	0.374
Birth weight (grams)	3259 (567)	3222 (504)	0.231
Vaginal delivery	421 (82.5)	628 (74.3)	<0.001
Obstetric complications *			
Overall PE	21 (4.0)	34 (4.0)	>0.999
Early-onset PE	3 (0.6)	2 (0.2)	0.377
Preterm PE	10 (1.9)	5 (0.6)	0.031
Term PE	11 (2.1)	29 (3.4)	0.187
SGA	49 (9.3)	97 (11.5)	0.242
SGA with PE	6 (1.1)	8 (0.9)	0.785
SGA without PE	43 (8.2)	89 (10.5)	0.187
Preterm birth	27 (5.1)	34 (4.0)	0.347
Gestational diabetes	40 (7.6)	56 (6.6)	0.514
Abruptio placentae	4 (0.8)	0 (0)	0.021
Stillbirth	4 (0.8)	1 (0.1)	0.074

* Data are given as mean (SD) or n (%). LMWH: low-molecular-weight heparin; GA: gestational age; PE: preeclampsia; SGA: small for gestational age.

## Data Availability

The data presented in this study are available on request from the corresponding author. The data are not publicly available due to privacy restrictions.
